# Distinct roles of spindle checkpoint proteins in meiosis

**DOI:** 10.1016/j.cub.2024.07.025

**Published:** 2024-07-29

**Authors:** Anuradha Mukherjee, Christos Spanos, Adele L. Marston

**Affiliations:** 1 The Wellcome Centre for Cell Biology, Institute of Cell Biology, University of Edinburgh, Edinburgh EH9 3BF, UK

## Abstract

Gametes are produced via meiosis, a specialized cell division associated with frequent errors that cause birth defects and infertility. Uniquely in meiosis I, homologous chromosomes segregate to opposite poles, usually requiring their linkage by chiasmata, the products of crossover recombination.^1^ The spindle checkpoint delays cell-cycle progression until all chromosomes are properly attached to microtubules,^2^ but the steps leading to the capture and alignment of chromosomes on the meiosis I spindle remain poorly understood. In budding yeast meiosis I, Mad2 and Mad3^BUBR1^ are equally important for spindle checkpoint delay, but biorientation of homologs on the meiosis I spindle requires Mad2, but not Mad3^BUBR1^.^3,4^ Here we reveal the distinct functions of Mad2 and Mad3^BUBR1^ in meiosis I chromosome segregation. Mad2 promotes the prophase to metaphase I transition, while Mad3^BUBR1^ associates with the TOGL1 domain of Stu1^CLASP^, a conserved plus-end microtubule protein that is important for chromosome capture onto the spindle. Homologous chromosome pairs that are proficient in crossover formation but fail to biorient rely on Mad3^BUBR1^-Stu1^CLASP^ to ensure their efficient attachment to microtubules and segregation during meiosis I. Furthermore, we show that Mad3^BUBR1^-Stu1^CLASP^ are essential to rescue the segregation of mini-chromosomes lacking crossovers. Our findings define a new pathway ensuring microtubule-dependent chromosome capture and demonstrate that spindle checkpoint proteins safeguard the fidelity of chromosome segregation both by actively promoting chromosome alignment and by delaying cell-cycle progression until this has occurred.

## Graphical abstract

**Graphical abstract:**
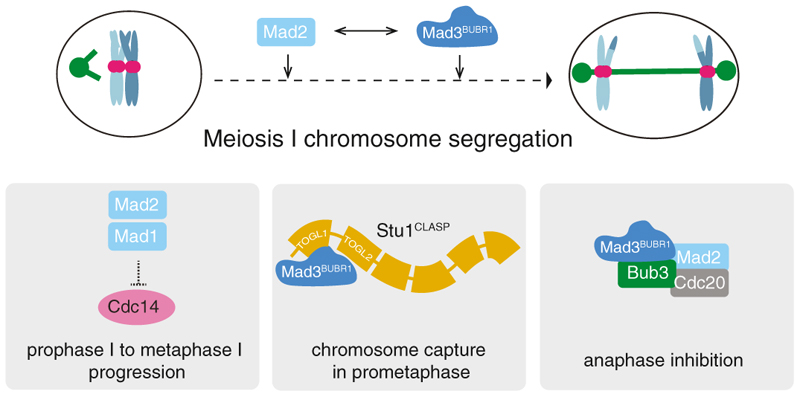

## Results and Discussion

### Distinct, checkpoint-independent functions for Mad2 and Mad3^BUBR1^ in meiosis I chromosome segregation

**Figure 1:**
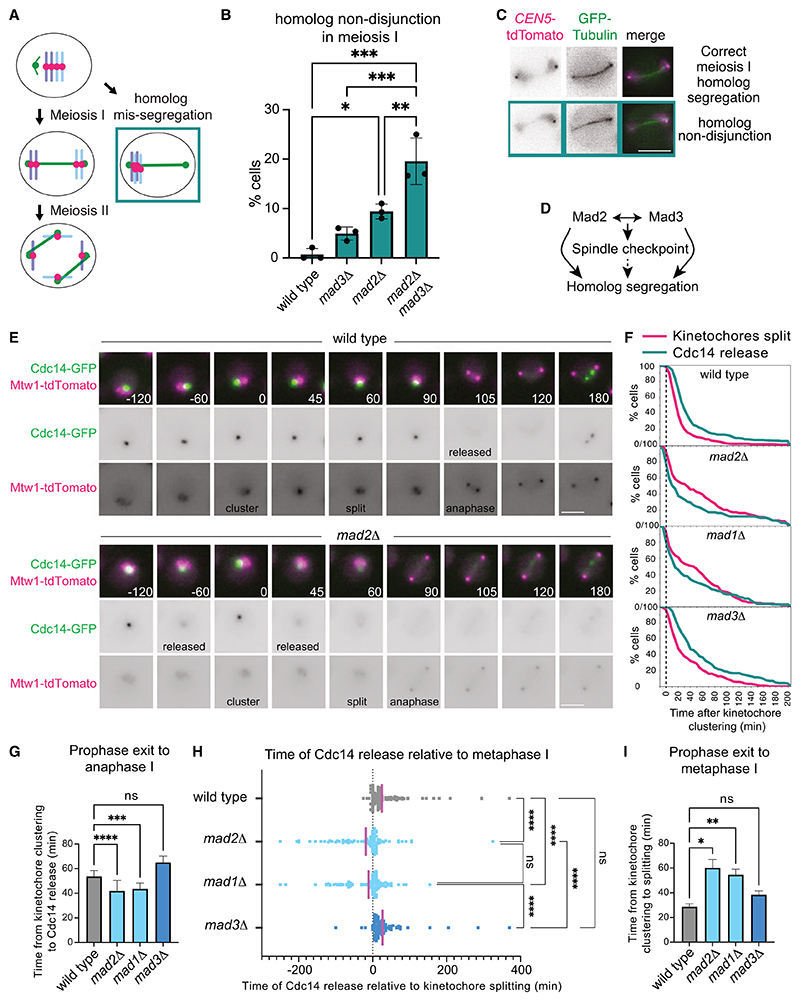
Mad2 and Mad3^BUBR1^ have distinct, spindle checkpoint-independent functions in meiosis I (A–C) *mad2Δ* and *mad3Δ* show additive effects on homolog segregation in meiosis I. Cells carrying *CEN5*-*tdTomato* and *GFP-TUB1* were induced to sporulate and live imaged. (A) Schematic of the experiment. (B) Percentage of cells of the indicated genotypes showing meiosis I non-disjunction. Mean of three biological repeats (*n* = 21–60, wild type; *n* = 56–68, *mad3Δ*; *n* = 53–65, *mad2Δ*; *n* = 57–60, *mad2Δ* *mad3Δ*). Error bars represent standard deviation, ****p < 0.0001, ***p ≤ 0.001, **p ≤ 0.01, *p ≤ 0.05, one way ANOVA (Tukey’s multiple comparisons test). (C) Representative images of correct meiosis I segregation and non-disjunction. Note that the *tetO* array can be visualized as a spot, while background TetR-tdTomato signal allows visualization of the nucleus. (D) Summary depicting the shared roles of Mad2 and Mad3 in the spindle checkpoint and separate roles in directing homolog segregation in meiosis I. (E–I) Mad2 orders cell-cycle events to promote the prophase to metaphase I transition. Live imaging of cells carrying Cdc14-GFP and Mtw1-tdTomato (kinet-ochores). (E) Representative images of wild-type and *mad2Δ* cells. (F) The percentage of cells with two kinetochore clusters and Cdc14 release was determined relative to the time of kinetochore clustering. (G) The mean time from kinetochore clustering to Cdc14 release was determined. ns, not significant; ****p < 0.0001, ***p ≤ 0.001, Kruskal-Wallis test. (H) Time of Cdc14 release relative to kinetochore splitting was plotted for individual cells. Magenta line indicates the mean. ns, not significant; ****p < 0.0001, one way ANOVA. (I) Mean time from kinetochore clustering to splitting. ns, not significant; **p ≤ 0.01, *p ≤ 0.05, Kruskal-Wallis test. For (F)–(I), number of cells scored was 209 (wild type), 99 (*mad2Δ*), 148 (*mad1 Δ*), and 156 (*mad3Δ*). Scale bars, 5 μm (C and E). See also Figures S1and S2.

The mitotic checkpoint complex (MCC) comprising Mad2-Mad3^BUBR1^-Bub3-Cdc20, an inhibitor of the anaphase promoting complex (APC^Cdc20^), is the canonical effector of the spindle checkpoint.^2^ Accordingly, both Mad2 and Mad3^BUBR1^ are required to impose a metaphase I delay in response to either unattached kinetochores or a lack of inter-homolog tension in meiosis.^3,4^ Furthermore, in an unperturbed meiosis, metaphase I and metaphase II are similarly shortened in the absence of either *MAD2*^5^ or *MAD3* or in the double mutant (Figures S1A–S1C). Although Mad3^BUBR1^ was suggested to delay meiotic prophase,^6^ the prophase marker, Zip1, a component of the synaptonemal complex, was present for a similar duration in wild-type, *mad2Δ*, *mad3Δ*, and *mad2Δ* *mad3Δ* cells (Figures S1D and S1E). Despite their shared role, Mad2 and Mad3^BUBR1^ also perform distinct functions in meiosis,^4,6^ and some mutations affecting kinetochore or spindle function show differential synthetic interactions with *mad2Δ* and *mad3Δ*.^7^ Consistently, Mad2, but not Mad3^BUBR1^, is required for homolog biorientation in an unperturbed meiosis^4^ (Figures S2A and S2B). Furthermore, we found that *mad2Δ* and *mad3Δ* have additive effects on the non-disjunction of homologs during meiosis I. Live imaging of meiotic cells with both chromosome V homologs labeled close to the centromere (*CEN5-tdTomato*) and carrying a spindle marker (*GFP-TUB1*) showed that homolog non-disjunction in meiosis I was only mildly elevated in *mad3 Δ* (~5%), reached ~10%–15% in *mad2Δ*, but was raised to ~20% in *mad2Δ* *mad3Δ* (Figures 1A–1C). Together, these observations reveal distinct spindle checkpoint-independent functions of Mad2 and Mad3^BUBR1^ in meiosis I (Figure 1D).

### Mad2 ensures orderly M phase events after prophase exit

In *C. elegans* germ cells and cultured human cells, Mad1-Mad2, but not BubR1(Mad3), ensure a timely G2-mitosis transition.^8^ Mad1-Mad2 curtails APC^Cdc20^ activity prior to mitosis independently of MCC formation to allow a threshold level of cyclin B to accumulate.^8^ Similarly, our live imaging of meiotic yeast cells carrying the kinetochore label Mtw1-tdTomato and the anaphase marker, Cdc14-GFP, showed that Mad1 and Mad2, but not Mad3^BUBR1^, promote the transition from prophase exit to metaphase I. Dispersed kinetochores in prophase re-cluster at prophase exit and split into two Mtw1-tdTomato clusters at metaphase I^9^ (Figure 1E). Cdc14-GFP phosphatase is released from the nucleolus in anaphase I and II to reverse M phase phosphorylation^10,11^ (Figure 1E). In wild-type and *mad3 Δ* cells, Cdc14 release invariably followed the splitting of the Mtw1-tdTomato cluster into two, reflecting the strict sequential order of metaphase I and anaphase I (Figures 1E and 1F). Surprisingly, however, in *mad1 Δ* and *mad2Δ* cells, Cdc14 release frequently occurred prior to the splitting of Mtw1-tdTomato (Figures 1E–1H). Consistent with their shorter metaphase I (Figure S1B), the time between prophase exit (kinetochore re-clustering) and anaphase I (Cdc14 release) was reduced in *mad1 Δ* and *mad2Δ* cells (Figure 1G). However, *mad1 Δ* and *mad2Δ*, but not *mad3Δ*, cells were delayed in reaching metaphase I after prophase exit (Figure 1I). Therefore, Mad1 and Mad2, but not Mad3^BubR1^, impose an order on M phase events and promote the timely transition between prophase and metaphase I. Premature Cdc14 activation leading to the untimely reversal of key phosphorylations and/or due to ectopic APC^Cdc20^ activity, as in *C. elegans*,^8^ could cause defective homolog biorientation in *mad2Δ* cells. However, bulk Rec8 loss occurs at anaphase I onset in *mad2Δ* cells (Figure S1B), indicating that not all cell-cycle events downstream of APC^Cdc20^ are disrupted in *mad2Δ* cells.

### Mad3^BUBR1^ specifically interacts with Stu1^CLASP^ independently of the spindle checkpoint

**Figure 2:**
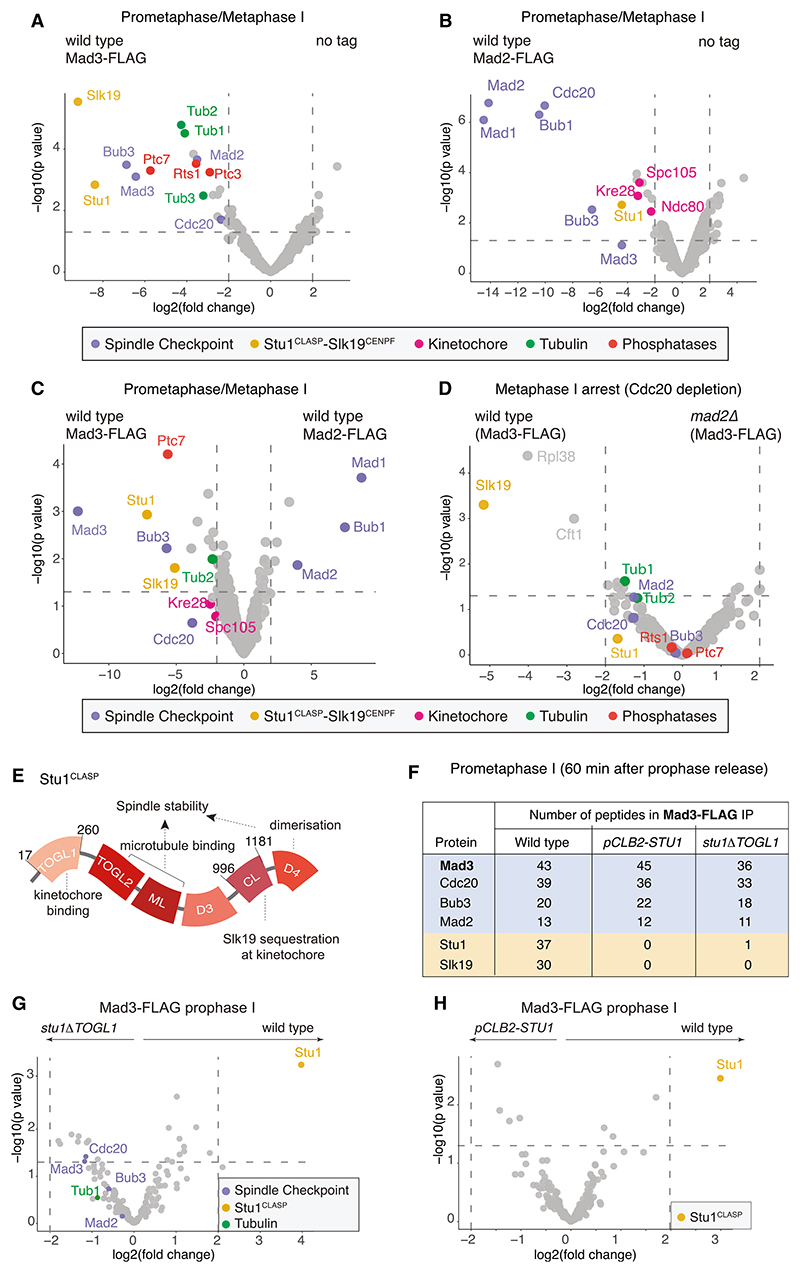
Mad3^BUBR1^ interacts with the TOGL1 domain of Stu1^CLASP^ (A–C) Immunoprecipitation and mass spectrometry of Mad2-FLAG and Mad3-FLAG during prometaphase/metaphase I. Volcano plots showing the relative enrichment of proteins immunoprecipitated with (A) Mad3-FLAG compared to no tag, (B) Mad2-FLAG compared to no-tag conditions, and (C) Mad3-FLAG versus Mad2-FLAG. Cells were harvested 75 min after release from a prophase I arrest (corresponding to prometaphase/metaphase I). (D) Mad3-FLAG interacts with Stu1 independently of the spindle checkpoint. Volcano plot showing the comparative enrichment of proteins identified by mass spectrometry in Mad3-FLAG immunoprecipitates from wild-type and *mad2Δ* cells harvested 6 h after inducing sporulation where progression beyond metaphase I was prevented by depletion of Cdc20 (*pCLB2-CDC20*). Rpl38 and Cft1 are likely contaminants. Results in (A)–(C) include data from three biological replicates, and (D) includes data from two biological repeats for each condition. Log_2_(fold change) between conditions is shown with corresponding *p* values. Dashed line indicates log_2_(fold change) = |2|. (E) Schematic of Stu1 protein with domains shown as identified by Funk et al.^17^ (F) List of proteins and their unique peptide counts as identified by one repeat of mass spectrometry after immunoprecipitation of Mad3-FLAG in the indicated strains. Note that all three strains have heterozygous *pCLB2-STU1*, with the other allele as indicated. Cells were harvested 60 min after release from prophase I. (G and H) Mad3 interaction with Stu1 is lost in *stu1ΔTOGL1* cells. Volcano plots after mass spectrometry showing the relative enrichment of proteins immunoprecipitated with Mad3-FLAG in (G) wild-type versus *stu1ΔTOGL1* and (H) wild-type versus *pCLB2-STU1* prophase I-arrested cells. In (A)–(D), (G), and (H), the absence of a colored dot for a kinetochore protein, phosphatase, or tubulin in the volcano plot means that it was not detected in this experiment. See also Figures S3 and S4 and Table S1.

We asked whether the differential functions of Mad2 and Mad3^BUBR1^ could be explained by interactions with different proteins. Immunoprecipitates from prometaphase/metaphase I (Figure S3A) wild-type cells carrying FLAG-tagged Mad3^BUBR1^ or Mad2 and a no-tag control were analyzed by mass spectrometry (Figures 2A–2C; Table S1). Mad2 predominantly bound Mad1, Bub1, and Cdc20, while Mad3^BUBR1^ was not enriched (Figure 2B), potentially indicating formation of a regulatory complex distinct from the MCC involved in promoting a timely prophase-meta-phase I transition (Figure 1I), similar to that described in *C. elegans*.^8^ In contrast, Bub3 is the major Mad3^BUBR1^ interactor, consistent with known direct binding events^12–15^ (Figure 2A). Proteins of the outer kinetochore, including Ndc80 and Spc105^KNL1^-Kre28, to which Bub1 binds directly as part of its role in the spindle checkpoint,^16^ were found only in Mad2 immunoprecipitates. In contrast, several distinct proteins, including tubulin subunits (Tub1, Tub2, and Tub3), three distinct phosphatases (Ptc3, Ptc7, and Rts1, a subunit of PP2A-B56), and the microtubule-regulator Stu1^CLASP^, along with its binding partner Slk19^CENPF^, were significantly enriched in the Mad3^BUBR1^ purification. Direct comparison confirmed that Stu1^CLASP^, Slk19^CENPF^, and Ptc7, in addition to Bub3, were most significantly enriched in Mad3^BUBR1^ over Mad2 purifications (Figure 2C). Since Mad3^BUBR1^ promotes homolog segregation independently of the spindle checkpoint or Mad2 (Figures 1A–1C), relevant interactions should persist upon spindle checkpoint inactivation. Comparison of Mad3-FLAG interactors with or without Mad2 (to abrogate the checkpoint) in Cdc20-depleted cells (metaphase I arrest) showed that while association of Slk19^CENPF^ with Mad3^BUBR1^ was greatly diminished in *mad2Δ* cells, the interactions with Stu1^CLASP^ and Ptc7 were maintained (Figure 2D). Stu1^CLASP^, but not Slk19^CENPF^, was also moderately enriched in Mad3^BUBR1^ immunoprecipitates from prophase I-arrested cells (Figure S3B), suggesting that Stu1 interacts with Slk19 only at prophase exit, potentially in response to check-point activity. We conclude that Mad3^BubR1^ associates with Stu1^CLASP^ independently of the spindle checkpoint and Mad2.

### Mad3^BUBR1^ interacts with Stu1^CLASP^ through its TOGL1 domain

Stu1^CLASP^ is a member of the conserved CLASP family of microtubule regulators that suppress catastrophes and promote rescue of plus ends to direct chromosome capture and alignment in mitosis.^18^ We reasoned that the N-terminal TOGL1 domain (Figure 2E), which mediates Stu1^CLASP^ localization to kinetochores in mitosis, but not its binding to microtubules or viability,^17^ might be relevant for Mad3^BUBR1^ function. We generated a version of Stu1^CLASP^ lacking the TOGL1 domain by deletion of amino acids 17–260, hereafter called Stu1ΔTOGL1. To circumvent detrimental effects of expressing only Stu1ΔTOGL1 in mitosis, these cells also carried *STU1* under control of the mitosis-specific *CLB2* promoter (*pCLB2-STU1*), which is shut off upon meiotic entry. Analysis of Mad3-FLAG immunoprecipitates in wild-type and *stu1ΔTOGL1* cells indicated that Stu1^CLASP^, but not Stu1ΔTOGL1, co-purified with Mad3^BUBR1^ in both prometaphase I and prophase I cells (Figures 2F–2H). Therefore, Mad3^BUBR1^ association with Stu1^CLASP^ requires its N-terminal TOGL1 domain. Slk19^CENP-F^ was also absent in Mad3-FLAG immunoprecipitates from *stu1ΔTOGL1* prometaphase I cells, indicating that the Mad3-Slk19^CENP-F^ interaction is likely to be indirect, via Stu1^CLASP^ (Figure 2F).

*STU1* is essential for microtubule organization in mitosis and viability.^19^ Although virtually all wild-type and *mad3Δ* cells underwent meiosis to produce four gametes, called a tetrad, over 75% of *pCLB2-STU1* cells failed to produce spores, with the remaining cells predominantly producing dyads (two spores), while 80% of *stu1ΔTOGL1* cells formed tetrads (Figure S3C). Therefore, although Stu1 is critical for sporulation, its N-terminal TOGL1 domain, which is required for Mad3^BUBR1^ association, is not. Anaphase I spindles were observed in live wild-type, *mad3Δ*, and *stu1ΔTOGL1* cells carrying *GFP-TUB1* (to label microtubules), but not in *pCLB2-STU1* cells (Figures S3D and S3E), indicating that Stu1 is required for bipolar spindle formation in meiosis I, similar to mitosis.^19^ Finally, purification of Stu1-GFP or Stu1Δ TOGL1-GFP revealed no major changes in interaction partners, including the retention of tubulin binding (Figures S3F–S3H), consistent with proficient meiotic spindle formation in *stu1Δ* *TOGL1* cells (Figures S3D and S3E). We note that Mad3^BUBR1^ was not recovered in either Stu1-GFP or Stu1ΔTOGL1-GFP immunoprecipitates (Figures S3F–S3H), indicating that only a minor fraction of cellular Stu1^CLASP^ interacts with Mad3^BUBR1^. Consistently, we found that Stu1 is around 8-to 10-fold more abundant than Mad3 in prophase and throughout the meiotic divisions in our recent whole-proteome dataset^20^ (Figure S3I). We conclude that *stu1ΔTOGL11* is a separation of functional allele that loses Stu1^CLASP^ interaction with Mad3^BUBR1^ while retaining its ability to organize microtubules.

### Stu1^CLASP^ kinetochore localization in meiosis does not require TOGL1

In mitosis, Stu1 is recruited to kinetochores via its TOGL1 domain, where it is particularly enriched when microtubules are not attached.^17^ However, Stu1ΔTOGL1-GFP localized similarly to wild-type Stu1-GFP in meiotic cells, being recruited to kinetochores at prophase exit before localizing also to the spindle in metaphase I and II, or the spindle midzone in anaphase I and II (Figures S4A and S4B). Quantification of the kinetochore localization at prophase I exit, when kinetochores cluster prior to spindle formation, revealed a small, but not statistically significant, decrease in Stu1ΔTOGL1-GFP compared to Stu1-GFP (Figures S4C and S4D). Stu1ΔTOGL1-GFP also localized to unattached kinetochores in meiotic cells treated with the microtubule-depolymerizing drug benomyl (Figure S4E), and its localization was independent of Mad3 (Figure S4F). It is unclear why the Stu1 TOGL1 domain is required for kinetochore localization in mitosis,^17^ but not meiosis. We speculate that the CL domain of Stu1^CLASP^ (Figure 2E), which contributes to kinetochore localization in mitotic cells,^17^ may be more important in meiosis, where kinetochores have a modified organization.^21^

### Mad3^BUBR1^ and the TOGL1 domain of Stu1^CLASP^ work together to promote meiosis I chromosome segregation

**Figure 3:**
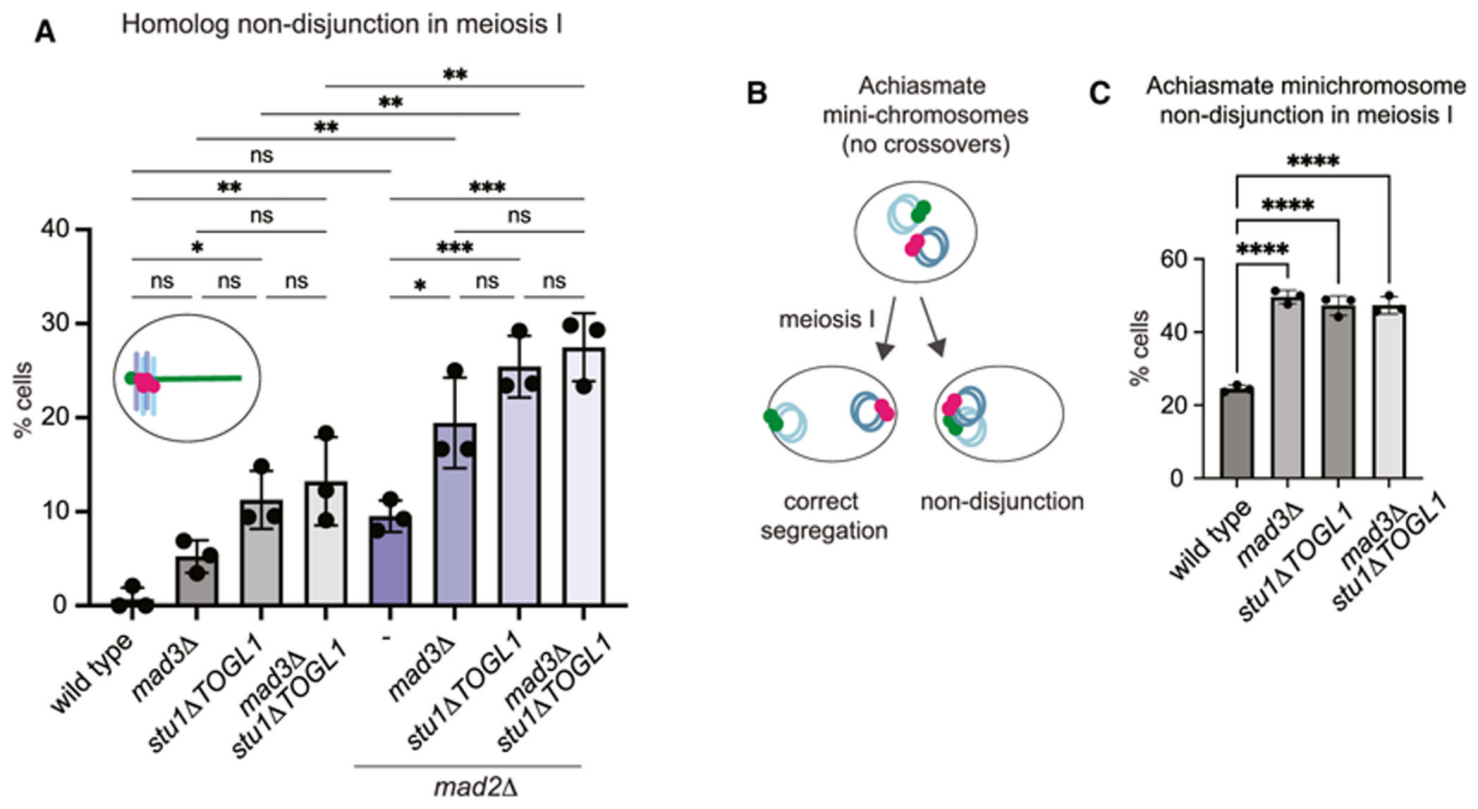
Mad3^BUBR1^ and the TOGL1 domain of Stu1^CLASP^ rescue the segregation of chromosomes that fail to cross over or biorient (A) Mad3-Stu1-TOGL1 and Mad2 act in distinct chromosome segregation pathways. Scoring of meiosis I non-disjunction after live imaging of endogenous homologous chromosomes competent for crossover recombination, as in Figures 1A–1C. Mean of three biological replicates (wild type, *mad3Δ*, *mad2Δ*, and *mad2Δ* *mad3Δ* are as in Figure 1B; *n* = 42–53, *stu1ΔTOGL1*; *n* = 49–60, *mad3Δ* *stu1ΔTOGL1*; *n* = 47–65, *mad2Δ* *stu1ΔTOGL1*; *n* = 57–60, *mad2Δ* *mad3Δ* *stu1ΔTOGL1*) where all genotypes were imaged concurrently is shown, with error bars representing standard deviation. ns, not significant; *p ≤ 0.05, **p ≤ 0.01, one way ANOVA (Tukey’s multiple comparisons test). (B and C) Segregation of achiasmate mini-chromosomes requires Mad3 and the TOGL1 domain of Stu1. (B) Schematic showing segregation of GFP-(green) and tdTomato-(red) labeled achiasmate chromosomes in meiosis I. (C) Non-disjunction of achiasmate mini-chromosomes in the indicated genotypes. For each of three biological replicates, 50 anaphase I cells (as judged by DAPI staining) were scored after fixation. Bar chart shows mean with error bars representing standard deviation. ****p ≤ 0.0001, one way ANOVA (Tukey’s multiple comparisons test).

We employed the *stu1ΔTOGL1* separation-of-function allele to test the function of the Stu1^CLASP^-Mad3^BUBR1^ interaction. *stu1ΔTOGL1* had no significant effect on the duration of prophase, metaphase I, or metaphase II, and *mad2Δ* and/or *mad3Δ* shortened metaphase I and II independently of *stu1Δ* *TOGL1* (Figure S1). Therefore, the TOGL1 domain of Stu1 is not required for the canonical spindle checkpoint. Next, we tested whether Mad3^BUBR1^ mediates meiotic chromosome segregation through Stu1^CLASP^, in which case meiosis I non-disjunction rates in *mad3Δ* *stu1ΔTOGL1* cells would be expected to be comparable to either single mutant. Homologs disjoined to the same pole in ~5% of *mad3Δ* cells, as described above, while this was increased to ~11% in *stu1ΔTOGL1* cells, though this difference was not statistically significant (*p* = 0.37; Figure 3A). Homolog mis-segregation in *mad3Δ* *stu1ΔTOGL1* cells was ~13%, similar to *stu1ΔTOGL1* alone (Figure 3A; *p* = 0.99). Therefore, although Stu1TOGL1 may have additional functions to Mad3^BUBR1^, the fact that *mad3Δ* does not increase homolog mis-segregation in *stu1ΔTOGL1* cells indicates that Mad3^BUBR1^ works in the same pathway as the TOGL1 domain of Stu1^CLASP^.

Since Mad3^BUBR1^ function in meiosis I chromosome segregation is most evident in the absence of *MAD2* (Figure 1B), we assessed the *stu1ΔTOGL1* mutant in the *mad2Δ* background. This revealed that *mad2Δ* exacerbated the meiosis I chromosome segregation defect in *mad3Δ* and *stu1ΔTOGL1* cells to a similar extent (Figure 3A). However, additional additive effects were not observed in the triple *mad2Δ* *mad3Δ* *stu1ΔTOGL1* mutant. Therefore, Mad3^BUBR1^ and the TOGL1 domain of Stu1^CLASP^ act in the same genetic pathway to promote meiosis I homolog segregation. In contrast, Mad2 acts in a distinct pathway (Figures 1B and 3A) and, unlike Mad3^BUBR1^, is important for homolog biorientation during meiosis I^4^ (see also below) and for ordering M phase events (Figures 1E–1I). Taken together, our findings indicate that Mad3^BUBR1^-Stu1^CLASP^ rescues the segregation of homologs that fail to biorient, while Mad2 functions in an independent pathway.

### The Mad3 and Stu1-TOGL1 pathway rescues the segregation of chromosomes that lack crossovers

Crossover recombination generates chiasmata that provide linkages between homologs, ensuring accurate meiosis I segregation.^3^ The occasional failure of a crossover can be tolerated, and so-called achiasmate or non-exchange chromosomes can undergo proper meiosis I segregation around 80% of the time, though the underlying mechanisms are not well understood.^22–26^ Interestingly, Mad3^BUBR1^, unlike Mad2, is essential for the segregation of such an achiasmate chromosome pair.^6^ Current models posit that the synaptonemal complex, which zips homologous chromosomes together as they recombine, persists at centromeres to maintain homolog pairing beyond prophase to rescue the segregation of chromosomes that fail to cross over.^27,28^ However, Mad3^BUBR1^ was found to act independently of centromere pairing and was instead proposed to mediate a prophase delay to allow achiasmate chromosome segregation.^6^ Because our live-cell imaging found no prophase delay in *mad3Δ* cells (Figure S1E), we instead hypothesized that Mad3^BUBR1^ directs achiasmate chromosome segregation via engaging Stu1^CLASP^. To test this idea, we introduced a pair of centromeric mini-chromosomes, one labeled with tdTomato (*tetO-*TetR-tdTomato), the other with GFP (*lacO-*GFP-LacI), into *mad3Δ*, *stu1ΔTOGL1*, and *mad3Δ* *stu1ΔTOGL1* cells (Figure 3B). Since the mini-chromosomes are both small and divergent in sequence, they will not cross over and therefore represent a pair of achiasmate chromosomes.^29^ In wild-type cells, red and green signal segregated to the same pole at anaphase I in 25% of cells, while segregation of the achiasmate mini-chromosomes was essentially random (~50%) in *mad3Δ* cells, as expected^6^ (Figure 3C). Crucially, *stu1ΔTOGL1* cells, where the Mad3^BUBR1^-Stu1^CLASP^ interaction is abolished (Figures 2F–2H), also exhibit random segregation of achiasmate mini-chromosomes in meiosis I, as does the *mad3Δ* *stu1ΔTOGL1* double mutant (Figure 3C). Therefore, the ability of Stu1^CLASP^ to bind Mad3^BUBR1^ is critical for achiasmate chromosome segregation.

### Mad3^BUBR1^ enables chromosome-spindle interactions through Stu1^CLASP^

**Figure 4:**
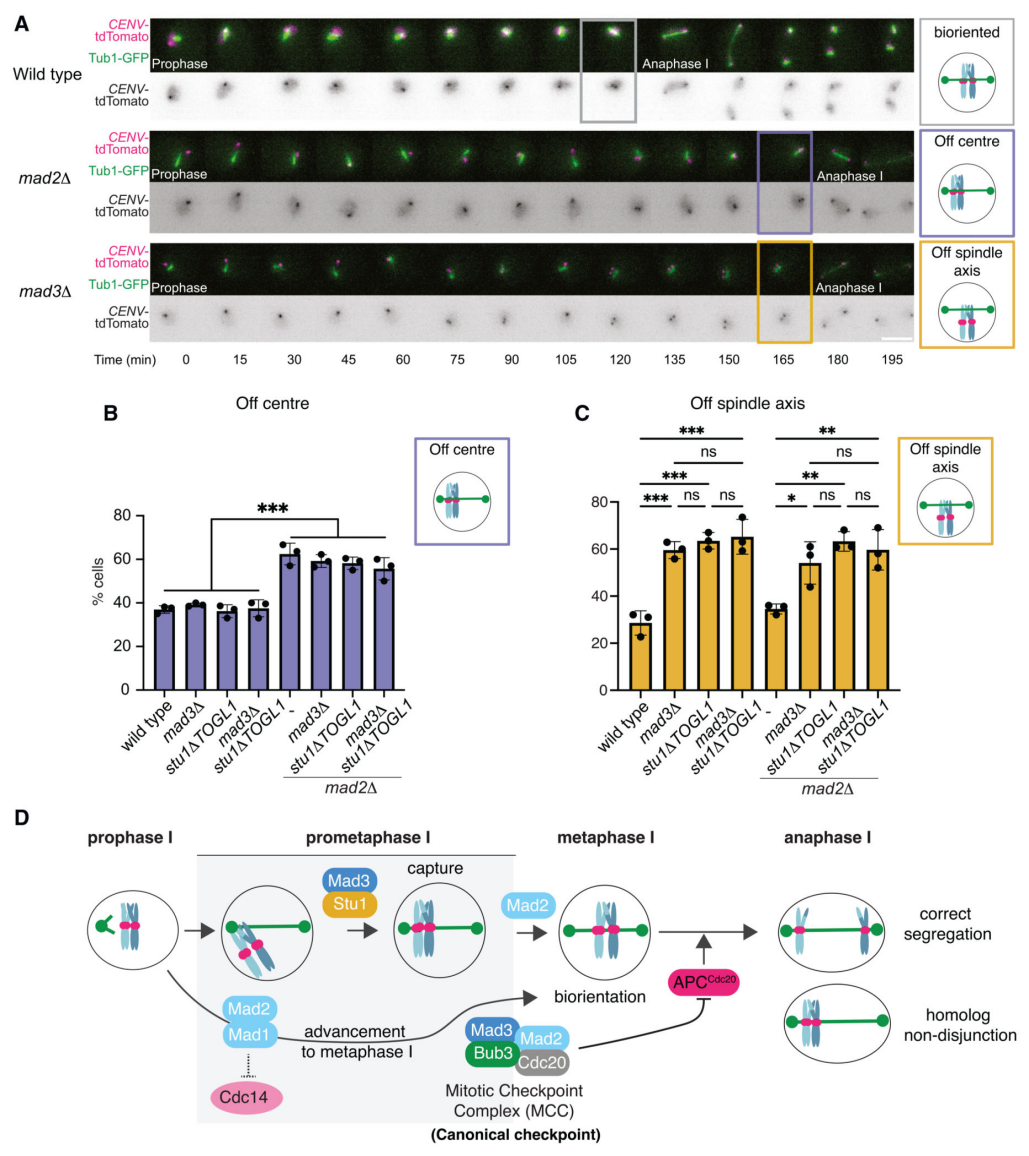
Mad3^BubR1^-Stu1^CLASP^ promote chromosome association with spindles during meiosis I (A–C) Live imaging reveals position and orientation of *CEN5-*tdTomato relative to the metaphase I spindle (GFP*-*Tub1). (A) Representative time series showing chromosome capture and alignment on the meiosis I spindle for the indicated genotypes and scenarios. Scale bar, 5 μm. (B and C) The percentage of cells where *CEN5*-tdTomato foci (which typically were observed as one or two foci) were located off the center of the spindle (B) or off the spindle axis (C) was scored in the last time point prior to anaphase I spindle elongation. Examples of correctly bioriented (gray box), off center (purple box), or off axis (yellow box) are shown in (A). Bar charts show mean of three biological replicates (*n* = 44–56, wild type; *n* = 52–62, *mad3Δ*; *n* = 36–52, *stu1ΔTOGL*; *n* = 41–49, *mad3Δ* *stu1ΔTOGL1*; *n* = 45–58, *mad2Δ*; *n* = 41–56, *mad2Δ* *mad3Δ*; *n* = 28–54, *mad2Δ* *stu1ΔTOGL1*; *n* = 25–58, *mad2Δ* *mad3Δ* *stu1ΔTOGL1*) with error bars representing standard deviation. ns, not significant; ****p < 0.0001, ***p ≤ 0.001, one-way ANOVA (Tukey’s multiple comparisons test). (D) Model for role of spindle checkpoint proteins in meiosis I chromosome segregation. Upon prophase exit, Mad1-Mad2 ensure Cdc14 phosphatase retention in the nucleolus to allow phosphorylation of key substrates important for progression to metaphase I. In prometaphase I, Mad3 engages Stu1 to facilitate chromosome association with microtubules. Also in prometaphase I, Mad2 ensures proper chromosome alignment in the center of the spindle through an unknown mechanism, potentially related to the earlier Mad2 function in preventing premature activation of Cdc14 phosphatase. Finally, in their canonical spindle checkpoint role, Mad2 and Mad3 assemble into the MCC to inhibit APC^Cdc20^ and delay anaphase I onset.

How might Stu1^CLASP^-Mad3^BUBR1^ contribute to the fidelity of chromosome segregation? Stu1 and CLASP proteins regulate microtubule dynamics at kinetochores to promote stable kinetochore capture and biorientation.^17,30–32^ Therefore, Mad3^BUBR1^ may promote kinetochore capture or biorientation via Stu1^CLASP^. To test this, we analyzed the position of *CEN5-*tdTomato foci relative to the metaphase I spindle prior to elongation at anaphase I (Figures 4A–4C). We considered instances where *CEN5*-tdTomato was located centrally on the spindle axis as “bioriented,” while asymmetric *CEN5*-tdTomato foci on the spindle axis were scored as “off center” and foci that did not co-locate with the GFP-Tub1 signal were scored as “off axis” (Figure 4A). Consistent with a previous report,^4^ *mad2Δ* showed defective biorientation, manifest as a significantly increased frequency of “off-center” *CEN5*-tdTomato foci (Figure 4B). However, compared to wild type, neither *mad3Δ* nor *stu1ΔTOGL1* exhibited defective biorientation unless *MAD2* was also deleted (Figure 4B). In contrast, the fraction of cells where *CEN5*-tdTomato was “off axis,” suggesting defective kinetochore capture by microtubules or stabilization of this attachment, was significantly increased over wild type in both *mad3Δ* and *stu1ΔTOGL1* mutants whether or not Mad2 was present (Figure 4C). These data show that Mad2 is important for positioning chromosomes in the center of the spindle axis (bio-riented), while Stu1^CLASP^-Mad3^BUBR1^ is important for chromosome association with the spindle. Therefore, Mad2 and Stu1^CLASP^-Mad3^BUBR1^ represent two distinct chromosome segregation pathways that respectively promote biorientation and chromosome-microtubule interactions, possibly through initial capture (Figure 4D).

## Conclusions

The spindle checkpoint prevents catastrophic segregation in response to improper kinetochore-microtubule attachments. Here we provide evidence that the components of this surveillance mechanism also contribute directly to the correction of improper or absent kinetochore-microtubule attachments. Incorporating these two key activities within the same proteins allows coordination of surveillance with segregation-promoting mechanisms (Figure 4D). Mad2 couples cell-cycle events as cells transition from prophase exit into mitosis, which could explain its role in promoting sister kinetochore biorientation, though the mechanism remains unclear. Mad3^BUBR1^ facilitates chromosome alignment through Stu1^CLASP^-dependent chromosome capture. We demonstrate that this pathway is critical to rescue chromosomes from lack of crossovers or a failure to biorient. Similar mechanisms may operate in mouse oocytes since Mad2 has a non-canonical function in curtailing APC activity at meiosis I exit^33^ and BUBR1 is required for robust kinetochore microtubule attachments.^34^ Safeguarding mechanisms such as those we identify here are therefore likely to play key roles in protecting against errors that lead to aneuploidy in human meiosis.

## Star⋆Methods

Detailed methods are provided in the online version of this paper and include the following:

- KEY RESOURCES TABLE
- RESOURCE AVAILABILITY
  - Lead contact
  - Materials availability
  - Data and code availability
- EXPERIMENTAL MODEL AND STUDY PARTICIPANT DETAILS
  - Yeast strains
  - Plasmids
- METHOD DETAILS
  - Meiotic induction and growth of yeast cultures
  - Sporulation efficiency assay
  - Mass spectrometry methods
  - Microscopy methods
- QUANTIFICATION AND STATISTICAL ANALYSIS

## Star⋆Methods

### Key Resources Table

**Table:** 

REAGENT or RESOURCE	SOURCE	IDENTIFIER
Antibodies		
Rat anti-tubulin	Bio-Rad	MCA77G; RRID: AB_325003
Donkey anti-rat FITC	Jackson ImmunoResearch	712-095-153; RRID: AB_2340652
Mouse anti-FLAG M2	Sigma-Aldrich	F1804; RRID: AB_262044
Mouse anti-GFP	Roche	11814460001; RRID: AB_390913
Chemicals, peptides, and recombinant proteins		
β-estradiol	Sigma	E2758
Benzonase	Merck Millipore (Novagen)	71206–3
Protein G Dynabeads	ThermoFisher	10009D
Chymostatin	Melford	C1104
Leupeptin (Hemisulphate)	Melford	L1001
E64	Melford	E1101
Pepstatin A	Melford	P2203
Antipain, dihydrochloride	Melford	A0105
Aprotinin	Melford	A2301
AEBSF hydrochloride 98%	ACROS Organics	32811010
N-Ethylmaleimidine 99+%	ACROS Organics	156100050
Complete-EDTA-free tablets	Roche	11873580001
Microcystin-L	LKT Laboratories	M3406
β-glycerophosphate	Pierce	90057
Bradford	Bio-Rad	5000001
NP-40	Fisher Scientific UK Ltd	13444269
DTT	Thermo-Fisher Scientific	R0861
β-mercaptoethanol	Merck Millipore	444203–250
Instant Blue	Abcam	ISB1L
ammonium bicarbonate	Sigma-Aldrich	09830
Trypsin	Merck Life Science UK	1084440001
Rapigest	Waters	186001861
Zymolyase	AMS Biotechnology	120491–1
Glusulase	Perkin Elmer	NEE154001EA
Polylysine	Novatein Biosciences	PE-54392
Deposited data		
Mass Spectrometry data	PRIDE: PXD048251	Perez-Riverol et al.^35^
Recombinant DNA		
*ARS1 CEN4 TRP1* plasmid containing *lacO*x224 arrays used for non-exchange chromosome segregation assay	This work	AMp1963
*ARS1 CEN5 URA3* plasmid containing *tetO*x112 arrays used for non-exchange chromosome segregation assay	Tanaka et al.^36^	p331 (AMp1973)
10kb *lacO* repeat in *LEU2* integrative vector	Straight et al.^37^	pAFS59 (AMp802)
*CEN4 TRP1* plasmid	Gietz et al.^38^	yCPlac22
Software and algorithms		
Max Quant analysis	Cox and Mann^39^	N/A
R studio	R	https://posit.co/download/rstudio-desktop
FIJI	National Institutes of Health	https://fiji.sc/
Prism 9	Graphpad	https://www.graphpad.com/features
Adobe Illustrator	Adobe	https://www.adobe.com/uk/

## Resource Availability

### Lead contact

Further information and requests for resources and reagents should be directed to and will be fulfilled by the lead contact, Adele Marston (adele.marston@ed.ac.uk).

### Materials availability

Yeast strains and plasmids used in this study can be obtained from the lead contact, without restriction. Yeast strains used in this study are given in Table S2.

## Experimental Model and Study Participant Details

### Yeast strains

All yeast strains are SK1 derivatives and are listed in Table S2. Gene deletions, promoter replacements and gene tags were introduced using standard PCR-based methods.^40^ *pCLB2-CDC20*,^41^ inducible-*ndt80 (pGAL1-NDT80, pGPD1-GAL4*.*ER*^42^), *CEN5*-GFP^43^ *CEN5-tdTomato*,^44^ *REC8-GFP*^44^, *ZIP1(700)-GFP*^45^, *CDC14-GFP*^44^, *MTW1-tdTomato*^44^, *GFP-TUB1*^44^ and *SPC42-tdTo-mato*^46^ were described previously. *stu1ΔTOGL1* was made by CRISPR-Cas9 in this study.

### Plasmids

Plasmids used in this study are listed in the key resources table. Plasmid AMp1963, used in the achiasmate minichromosome assay and which carries *CEN4, TRP1* and *lacO* arrays, was generated by restriction digest of yCPlac22 and pAFS59 with *Sal*I and *Kpn*I and ligating the respective 10,108bp *lacO* and 4,743 bp *CEN4 TRP1* fragments.

## Method Details

### Meiotic induction and growth of yeast cultures

Diploids were recovered from 20% glycerol stock at −80°C onto YPG plates (1% yeast extract, 2% bactopeptone, 2% glycerol, and 2% agar) and grown for 12h. They were patched on 4% YPDA agar plates (1% yeast extract, 2% bactopeptone, 4% glucose, and 2% agar) for 6-8h, then inoculated in YPDA medium (1% yeast extract, 2% bactopeptone, 2% glucose, 0.3 mM adenine) and shaken at 250 rpm at 30°C for approximately 24h before being diluted to OD_600_ = 0.2–0.4 in YPA (1% yeast extract, 2% peptone, 1% potassium acetate) and grown for another 12-16h. In the morning, cells were pelleted and washed with sterile dH_2_O before being resuspended in SPO media (0.3% potassium acetate) at OD_600_ = 1.8–1.9 and shaken at 250 rpm at 30°C for the duration of the experiment. Cells grown for live-cell imaging were resuspended at OD_600_ = 2.3 and for mass spectrometry experiments at OD_600_ 2.5 in SPO media. For synchronous meiosis, inducible-*NDT80* was used to allow prophase I block-release.^47^ For prophase I arrest, cells with inducible-*NDT80* were harvested after 6h in SPO medium. For metaphase I arrest, cells with *pCLB2-CDC20* were harvested after 6h in SPO medium.

### Sporulation efficiency assay

Diploids were recovered on YPG plates overnight and sporulated in liquid SPO media at 250 rpm at 30°C for 72h and 200 cells were scored by light microscopy to determine the proportion of triads and tetrads, dyads and non-sporulated cells.

### Mass spectrometry methods

#### Conjugating anti-FLAG or anti-GFP to dynabeads

Protein G Dynabeads (Invitrogen) were washed twice in 1mL 0.1M Na-phosphate, pH 7.0, before incubating with 1/10^th^ volume of M2 anti-FLAG monoclonal antibody (Sigma) or 1/5^th^ volume of anti-GFP antibody (Roche) and 50μL of 0.1M Na-phosphate with gentle agitation for 30 min at room temperature. Beads were washed twice in 1mL of 0.1M Na-phosphate pH 7.0 with 0.01% Tween 20, then washed twice with 1mL of 0.2 M triethanolamine, pH 8.2. Antibody-conjugated Dynabeads were resuspended in 1mL of 20mM DMP (Dimethyl Pimelimidate, D8388, Sigma) in 0.2M triethanolamine, pH8.2 (prepared immediately before use) and incubated with rotational mixing for 30 min at room temperature. Beads were concentrated, the supernatant removed and 1mL of 50mM Tris-HCl, pH7.5 added before incubating for 15min with rotational mixing. The supernatant was removed and beads were washed three times with 1mL 1XPBST+0.1% Tween 20 before resuspending in 300mL of 1xPBST.

#### Immunoprecipitation

Either 3L (Figures 2A–2D) or 200mL (Figures 2F–2H, S3B, and S3F–S3H) of meiotic culture grown at OD_600_ = 2.5 was harvested and washed once with sterile dH_2_O. Cells were pelleted and resuspended in 20% v/w 2 mM PMSF and snap frozen as small ‘noodles’ by releasing drops of cells into liquid nitrogen. These noodles were filled in metal canisters pre-cooled in liquid nitrogen and cells lysed by 5 rounds of 30/s speed for 3 min each in the twin bio-pulverizer Retsch MM400. Grindate was then emptied out of the canisters into a 50mL falcon tube and stored at −80°C. For immunoprecipitation, the cryogrindate was thawed and resuspended in 20% w/v H0.15M lysis buffer (25mM HEPES pH8, 2mM MgCl_2_, 0.1mM EDTA pH8.0, 0.5mM EGTA-KOH pH8.0, 15% glycerol, 0.1% NP-40, 150mM KCl) with phosphatase and protease inhibitors (CLAAPE, comprising 10 μg/mL each of chymostatin, leupeptin, antipain, pepstatin and E64, together with 2mM AEBSF, 0.8mM Na Orthovanadate, 0.2uM microcystin, 1x EDTA-free Roche protease inhibitor tablet, 2mM NEM, 4mM β-glycerophosphate, 2mM Na pyrophosphate, 10mM NaF). 40U/ml of Benzonase (Novagen) was added to the lysate and incubated for 1h at 4falcon tube and stored 4°C with rotation to digest DNA. Samples were centrifuged for 10 min at 4000 rpm at 4°C and supernatant was collected in new pre-chilled falcon tubes. Protein concentration was determined by Bradford assay, and each lysate was adjusted to the same volume and protein concentration. 50μL of each adjusted lysate was added to 10μL 4xLDS + 5% β-mercaptoethanol, boiled at 95°C for 5min and stored at −20°C as input. 2μg α-GFP (Roche) or 0.05 μg α-FLAG (Sigma) previously conjugated to Protein G-dynabeads were added to each sample and incubated with rotation at 4°C for 2.5h. Dynabeads were concentrated using a pre-chilled magnet and the flow through was discarded. The beads were transferred into an eppendorf tube and washed once with buffer H0.15M with inhibitors and 2mM DTT, then three more times with buffer H0.15M with inhibitors. Beads were concentrated on the magnet, resuspended in 50μL 1xLDS + 5% β-mercaptoethanol and boiled at 70°C for 10min to elute. Samples were spun down at 13,200rpm for 5min before the eluate was transferred to a fresh eppendorf tube and stored at −20°C indefinitely for preparation for mass spectrometry.

#### In gel digestion of protein samples for mass spectrometry

In-gel digestion was used to prepare samples for mass spectrometry in Figures 2A–2D and 2F. Yeast growth conditions and the immunoprecipitation protocol used was the same as above, with a few modifications. 3L of SPO cultures at OD_600_ = 2.5 were harvested and 500μL Protein G dynabeads previously conjugated to 50μL M2 α-FLAG antibody were added to each extract which was made from approximately 15g of cryogrindate. Proteins were eluted from beads in 100μL 1xLDS + 5% β-mercaptoethanol, out of which 90μL was loaded on NuPAGE Novex 4-12% Bis-Tris Gel (Life Technologies) gels and run for 6min so that all proteins enter the gel. The gel was stained by incubating with agitation in Instant Blue (Abcam) and washed three times for 5min each with dH_2_O. Protein bands were cut from the gel and chopped into ~1mm^3^ pieces using a new clean scalpel, and the pieces were collected in an eppendorf tube. The pieces were submerged in 50mM ammonium bicarbonate (ABC) for 30min. ABC was discarded and 100% acetonitrile (ACN) was added until gel pieces were submerged and incubated for another 30min. ~80ul 10mM DTT in 50mM ABC was added to the gel pieces and incubated for 30 min at 37°C. DTT solution was removed and gel pieces were resuspended in ACN for 5min and any excess liquid was removed. ~80ul 55mM iodoacetamide dissolved in ABC was added to cover the pieces and incubated in the dark at RT for 20mins. The liquid was removed and gel pieces were incubated with 50mM ABC buffer for 5 min at 37°C, the ABC was removed, and then the gel pieces were incubated in ACN for 5 mins at 37°C. All liquid was removed and enough trypsin digestion mix (0.013 μg/ml trypsin, 10% ACN, 10mM ABC) was added to cover the gel pieces and left initially at 4°C and then at 37°C overnight for 12-15h in a moist chamber. 0.1% or 10% TFA was added to the gel pieces in trypsin digestion mix to stop over-digestion of peptides and the solution was kept at room temperature for 15min to allow all peptides to diffuse out form the gel. 1μL of sample was dropped on a pH paper to confirm that the solution has pH < 2.0.

#### Filter-aided sample preparation (FASP) of protein samples

FASP was used to prepare samples for mass spectrometry in Figures 2G, 2H, S3B, and S3F–S3H, as described,^48^ with a few modifications. Proteins were eluted from beads by incubating in 30μL 0.1% Rapigest (Waters) dissolved in 50mM ABC at 37°C for 30min, removing the eluate and then repeating to obtain a further 30μL of eluate. Pooled eluates from the two elutions were stored at −20°C. On the day of trypsin digestion, 10% volume of 1M DTT was added to samples and boiled for 5 min at 95°C with agitation. Tubes were cooled to room temperature before adding 3x vol of 8M urea in 100mM Tris-HCl pH8.0 (UBB) to each sample. The whole sample was transferred onto Sartorius Stedim Biotech’s Vivacon 500 MWCO 30 000 VN01H21 column and spun down at 10,000rpm for 10-15 min at room temperature to bind all peptides to the membrane. 100μL of 55mM iodoacetamide dissolved in UBB was added, the tube shaken at 600rpm for 1 min at RT in a theromixer, and incubated in the dark for 30min before spinning the buffer through the column. The column was then washed once with 100μL UBB and twice with 100μL ABC. The column was completely dried before adding 60μL of trypsin digestion mix (0.013 μg/ml trypsin, 0.002% TFA, 50mM ABC) onto the column membrane. Columns were capped and sealed with parafilm before being shaken at 600rpm for 1 min at room temperature and then incubated at 37°C overnight for ~15h in a moist chamber. Parafilm was removed and the columns were centrifuged to elute trypsin-digested peptides into new protein protein LoBind tubes containing 10μL of 10% TFA to stop the trypsin digestion. 1μL of sample was dropped on a pH paper to confirm that the solution was pH < 2.0.

#### Mass spectrometry

Stage tips were prepared by inserting three Empire C18 disks (3M) inside a p200 pipette tip. 20μL MeOH and 50μL 0.1% TFA was passed through the tip to calibrate the disks at the correct pH. All the liquid from the gel digestion or the in-column digestion was passed through the stage tip by microfuging for ~10min. The tip was then washed again with 0.1% TFA and stored at −20°C. Peptides were eluted in 40 μL of 80% acetonitrile in 0.1% TFA and concentrated down to 1 μL by vacuum centrifugation (Concentrator 5301, Eppendorf, UK). The peptide sample was then prepared for LC-MS/MS analysis by diluting it to 6 μL by 0.1% TFA.

All LC-MS analyses were performed on an Orbitrap Fusion Lumos Tribrid Mass Spectrometer (Thermo Fisher Scientific, UK) both coupled on-line, to an Ultimate 3000 HPLC (Dionex, Thermo Fisher Scientific, UK). Peptides were separated on a 50 cm (2 μm particle size) EASY-Spray column (Thermo Scientific, UK), which was assembled on an EASY-Spray source (Thermo Scientific, UK) and operated constantly at 50°C. Mobile phase A consisted of 0.1% formic acid in LC-MS grade water and mobile phase B consisted of 80% acetonitrile and 0.1% formic acid. Peptides were loaded onto the column at a flow rate of 0.3 μL min^-1^ and eluted at a flow rate of 0.25 μL min^-1^ according to the following gradient: 2 to 40% mobile phase B in 150 min and then to 95% in 11 min. Mobile phase B was retained at 95% for 5 min and returned back to 2% a minute after until the end of the run (190 min). Survey scans were recorded at 120,000 resolution (scan range 350-1500 m/z) with an ion target of 4.0e5, and injection time of 50ms. MS2 was performed in the ion trap at a rapid scan mode, with ion target of 2.0E4 and HCD fragmentation^49^ with normalized collision energy of 27. The isolation window in the quadrupole was 1.4 Thomson. Only ions with charge between 2 and 6 were selected for MS2. Dynamic exclusion was set at 60s.

#### Analysis of mass spectrometry data

The MaxQuant software platform^39^ version 1.6.1.0 was used to process the raw files and search was conducted against the complete/reference proteome set of *Saccharomyces cerevisiae* SK1 strain (combined Saccharomyces Genome Database and in-house database - released in August 2019), using the Andromeda search engine.^50^ For the first search, peptide tolerance was set to 20 ppm while for the main search peptide tolerance was set to 4.5 p.m. Isotope mass tolerance was 2 ppm and maximum charge to 7. Digestion mode was set to specific with trypsin allowing maximum of two missed cleavages. Carbamidomethylation of cysteine was set as fixed modification and oxidation of methionine, was set as variable modification. Label-free quantitation analysis was performed by employing the MaxLFQ algorithm as described.^51^ Absolute protein quantification was performed as described.^52^ Peptide and protein identifications were filtered to 1% FDR.

Statistics from LFQ data were processed using Bioconductor *DEP* R package according to Zhang et al.^53^ (https://github.com/arnesmits/DEP).

### Microscopy methods

#### Live cell imaging

To adhere cells, 5μL of ConA (5 mg/mL ConcanavalinA in 50mM CaCl_2_, 50mM MnCl_2_) was spread at the bottom of chambers in 8-well glass-bottomed Ibidi dish (Thistle Scientific) using a plastic loop and incubated at 30°C for 15min. ConA was aspirated and the chambers washed three times with 500μL sterile dH_2_O and stored in the dark. To prepare cells for imaging, 10mL of meiotic cultures were started at OD_600_ = 2.3 in SPO media. After 3h, 1mL of culture was spun down at 3000rpm for 1min. The pellet was resuspended in 300μL SPO media, added to the Ibidi dish and incubated for 20 min at 30°C. Wells were washed with 500μL SPO media twice before adding 400μL fresh SPO media. For the inducible Ndt80 block-release system, 200μL SPO was added while setting up the Ibidi dish, and another 200μL SPO with 2μM β-estradiol was added immediately before starting the time lapse imaging. For depolymerizing microtubules in Figure S6E, benomyl was pre-dissolved in SPO medium and was added at a final concentration of 50 μg/ml to the wells along with β-estradiol. Fluorescent microscopy was performed using Zeis Axioplan 2 microscope with 100x Plan ApoChromat NA 1.4 oil lens. Images were acquired through ORCA FLASH 4 CCD camera with auto-focus operated through Axio-vision software and with 2x2 binning. GFP-Tub1 was imaged at 4% laser intensity for 80ms, *CEN5*-tdTomato was imaged at 4% intensity for 100ms, Mtw1-tdTomato was imaged at 10% intensity for 100ms or 150ms. Rec8-GFP was imaged at 5% intensity for 100 ms. Zip1-GFP was imaged at 1.5% intensity for 50 ms. Spc42-tdTomato was imaged at 5% intensity for 200 ms. Cdc14-GFP was imaged at 5% intensity for 100 ms. For all fluorescent channels, 9 z-slices of 0.7μm interval were captured. Brightfield was used for auto-focus and imaged only for the middle slice with 3V for 10ms. Chromosome segregation, metaphase I/II timing and Cdc14-GFP assays were imaged every 15min. Prophase timing (Zip1-GFP) was imaged every 8 min. Biorientation assays were imaged every 5min for 10h in total.

#### Image analysis

ImageJ software (National Institutes of Health) was used to max project the z-stacks and for visualising the images. To quantify signal intensity in Figure S6D, A circular region was drawn encompassing the region of GFP and tdTomato signal overlap, and the ratio of integrated density measurement of the GFP signal over the tdTomato signal was calculated. Final image assembly was conducted in Adobe Illustrator.

#### Imaging of GFP-labelled chromosomes in fixed cells

For the chromosome segregation assay reported in Figure S2, 150μL meiotic culture at OD_600_ = 1.9 in SPO media was added to 15μL of 37% v/v formaldehyde in 1.5mL Eppendorf tubes and fixed for 8 min at room temperature. Tubes were then spun at 13,200rpm for 1min, supernatant removed and resuspended in 1mL 80% EtOH. Tubes were spun again for 30s, EtOH poured out, spun again for 15s and the remaining EtOH removed with a pipette. The pellet was resuspended in 20μL of 1 μg/ml DAPI and temporarily stored at 4°C for up to one week. 3μL of cells were placed on a Superfrost microscope slide (Thermo Fisher Scientific), covered with a coverslip (VWR) and sealed with nail polish. The coverslip was pressed tightly against the slides and imaged on an Axioplan 2 microscope with 100x Plan ApoChromat NA 1.4 oil lens with 5% GFP, 10% tdTomato and 2% DAPI to visualize GFP and tdTomato dots and DNA.

#### Achiasmate minichromosome segregation assay

Diploid cells carried homozygous *pURA3-GFP-LacI* and heterozygous *pURA3-TetR-tdTomato* integrated into the genome, and AMp1963 and AMp1973 plasmids. The plasmids were maintained by selecting on synthetic complete glucose agar lacking both uracil and tryptophan (SD/-ura/-trp), before cells were inoculated consecutively in YPDA, YPA and SPO liquid media as described above for induction of meiosis and sporulation. Samples were collected 2h after inducing sporulation and every 30min thereafter until 4:30h. Cells were fixed and processed as described above “Imaging of GFP-labelled chromosomes in fixed cells“, stored in 4°C and were counted on the same day. Only cells with a single GFP and single tdTomato focus at the binucleate stage were included in segregation scoring.

#### Immunofluorescence

200μL of meiotic culture was centrifuged at 13,200rpm for 1min and resuspended in 500μL of 3.7% v/v formaldehyde in 0.1M KPi, pH6.4 (potassium phosphate buffer: 27.8mM K_2_HPO_4_ and 72.2mM KH_2_PO_4_) to fix overnight at 4°C. Cells were then spun down and washed three times with 1mL of 0.1M KPi buffer, and resuspended in 1mL of sorbitol-citrate (1.2M sorbitol, 0.1M KH_2_PO_4_, 36mM citric acid). Cells were spun down again and resuspended in digestion mix (200μL sorbitol-citrate, 20μL glusulase and 6μL 10 mg/ml zymolase) and incubated at 30°C for 2h or until cells become phase-dark under light microscope. Once digested, cells were pelleted, washed with 1mL sorbitol-citrate and then resuspended in ~50μL sorbitol-citrate. 5μL of 0.1% polylysine was added to each well of multi-well slides (Thermo Fisher Scientific) for 5 min at room temperature before being rinsed with dH_2_O and air-dried. 5μL of digested cells were added to each well and incubated for 10min, before aspiration and submerging in MeOH for 3min followed by acetone for 10s. 5μL of Rat α-tubulin (AbD Serotec) primary antibody diluted in 1:50 in PBS-BSA (1% w/v BSA, 0.04M K_2_HPO_4_, 0.01M KH_2_PO_4_, 0.15M NaCl, 0.1% w/v NaN_3_) was added to each well and incubated in a moist chamber for 1h at room temperature. Primary antibody was aspirated and wells washed five times each with 5μL of PBS-BSA. 5μL of Donkey anti-rat-FITC (Jackson ImmunoResearch) secondary antibody was added, and slides were incubated in a dark moist chamber for 1h, then each well was washed five times with 5μL of PBS-BSA. 3μL of DAPI-mount (9mM p-phenylenediamine, 0.04M K_2_HPO_4_, 0.01M KH_2_PO_4_, 0.15M NaCl, 0.1% w/v NaN_3_, 50 ng/ml DAPI, 90% v/v glycerol) was added to each well, before the slide was covered with a glass coverslip and sealed with nail paint. Slides were stored at −20°C and visualized on a Zeiss Axioplan 2 microscope with 100x Plan ApoChromat NA 1.4 oil lens.

## Quantification and Statistical Analysis

Statistical analysis and graphs were generated using Graphpad Prism 9 software (San Diego). Micrographs and graphs were assembled using Adobe Illustrator. Statistical details of all experiments are given in the figure legends.

## Supplementary Material

Supplemental information Supplemental information can be found online at https://doi.org/10.1016/j. cub.2024.07.025.

Table S2- yeast strains

## Data Availability

Mass spectrometry datasets reported in this study have been deposited at PRIDE with the accession number PXD048251. reviewer_pxd048251@ebi.ac.uk The paper does not report original code. Any additional information required to reanalyze the data reported in this paper is available from the lead contact upon request.

## In brief

Mukherjee et al. identify differential roles of Mad2 and Mad3^BUBR1^ in meiosis, outside their canonical functions in delaying anaphase onset as part of the spindle checkpoint. While Mad2 promotes meiotic progression prior to metaphase I, Mad3^BUBR1^ interacts with the microtubule regulator Stu1^CLASP^ to promote chromosome capture onto the spindle.

## Highlights

- Spindle checkpoint proteins Mad2 and Mad3^BubR1^ play distinct roles in meiosis I
- Mad2 ensures prophase I to metaphase I progression and homolog biorientation
- Mad3^BubR1^ interacts with the TOGL1 domain of Stu1^CLASP^ to capture chromosomes
- Mad3^BubR1^-Stu1^CLASP^ rescues the segregation of homologs that fail to cross over
