# Supplementary material for: Distinct roles of spindle checkpoint proteins in meiosis
Source: Curr Biol. Author manuscript; Available in PMC 2025 Apr 10. (PMC7617576; doi:10.1016/j.cub.2024.07.025)
Supplement: Supplemental information — can be found online at https://doi.org/10.1016/j. cub.2024.07.025. [file EMS204298-supplement-Supplemental_information.pdf]

Current Biology, Volume *34*

Supplemental Information

Distinct roles of spindle checkpoint proteins in meiosis

Anuradha Mukherjee, Christos Spanos, and Adele L. Marston

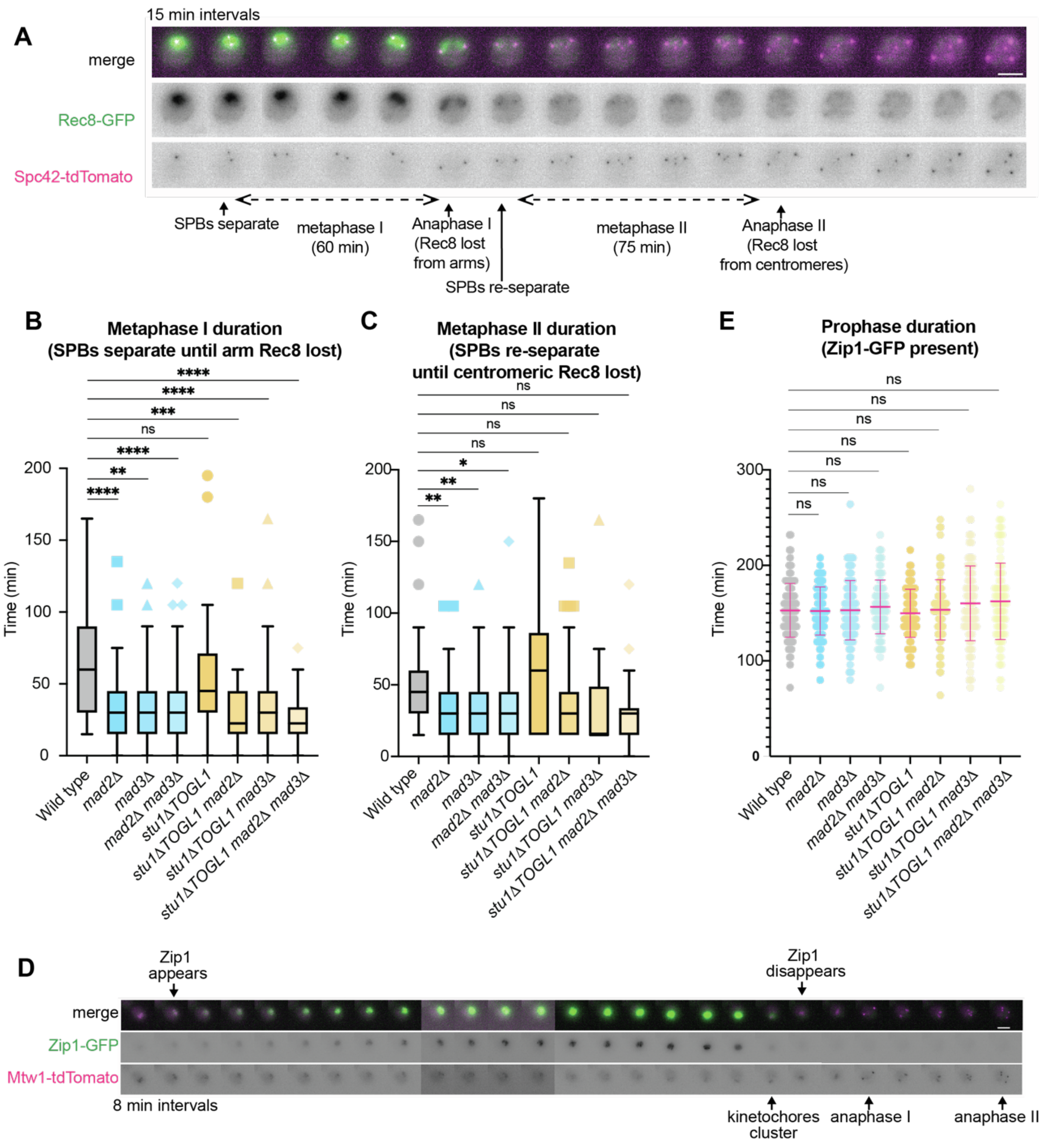

Figure S1 Mad2 and Mad3 extend the duration of metaphase I and II in an unperturbed meiosis, but do not affect the duration of meiotic prophase. Related to Figure 1.

(A-C) Live cell imaging of Rec8-GFP and Spc42-tdTomato (A) Example image of a wild type cell with key transition points labelled. Images were captured every 15 min. Scale bar = 5µm. (B) Metaphase I duration was determined as the time interval between SPB splitting until Rec8 loss from chromosome arms. (C) Metaphase II duration was scored as the time interval between SPB re-splitting until centromeric Rec8 loss. In B and C, Tukey box and whiskers plots are shown with the horizontal line representing the median. The number of cells scored was 51 (wild type), 53 (*mad2Δ*), 40 (*mad3Δ*), 54 (*mad2Δ mad3Δ*), 48 (*stu1ΔTOGL1*), 49 (*stu1ΔTOGL1 mad2Δ*), 22 (*stu1ΔTOGL1 mad2Δ*) and 22 (*mad2Δ mad3Δ stu1ΔTOGL1*). ****

p<0.0001, ***p≤0.001, **p≤0.01, *p≤0.05, ns, not significant, Krustal-Wallis test. Note that capturing images every 15 min leads to poor temporal resolution at metaphase II which could account for the fact that the differences between wild type and the *stu1ΔTOGL1* strains in metaphase II do not reach statistical significance. (D and E) Live cell imaging of Zip1-GFP and Mtw1-tdTomato, with images captured every 8 min. Scale bar = 5μm. (D) Representative imaging of a wild-type cell, with the key events indicated. (E) The time interval between Zip1 appearance and disappearance was scored for the indicated genotypes. The magenta horizontal line represents the mean and the bars represent standard deviation. ns, not significant, Ordinary One way Annova. Number of cells scored was 113 (wild type), 104 (*mad2Δ*), 113 (*mad3Δ*), 103 (*mad2Δ mad3Δ*), 104 (*stu1ΔTOGL1*), 101 (*stu1ΔTOGL1 mad2Δ*), 108 (*stu1ΔTOGL1 mad3Δ*) and 109 (*stu1ΔTOGL1 mad2Δ mad3Δ*).

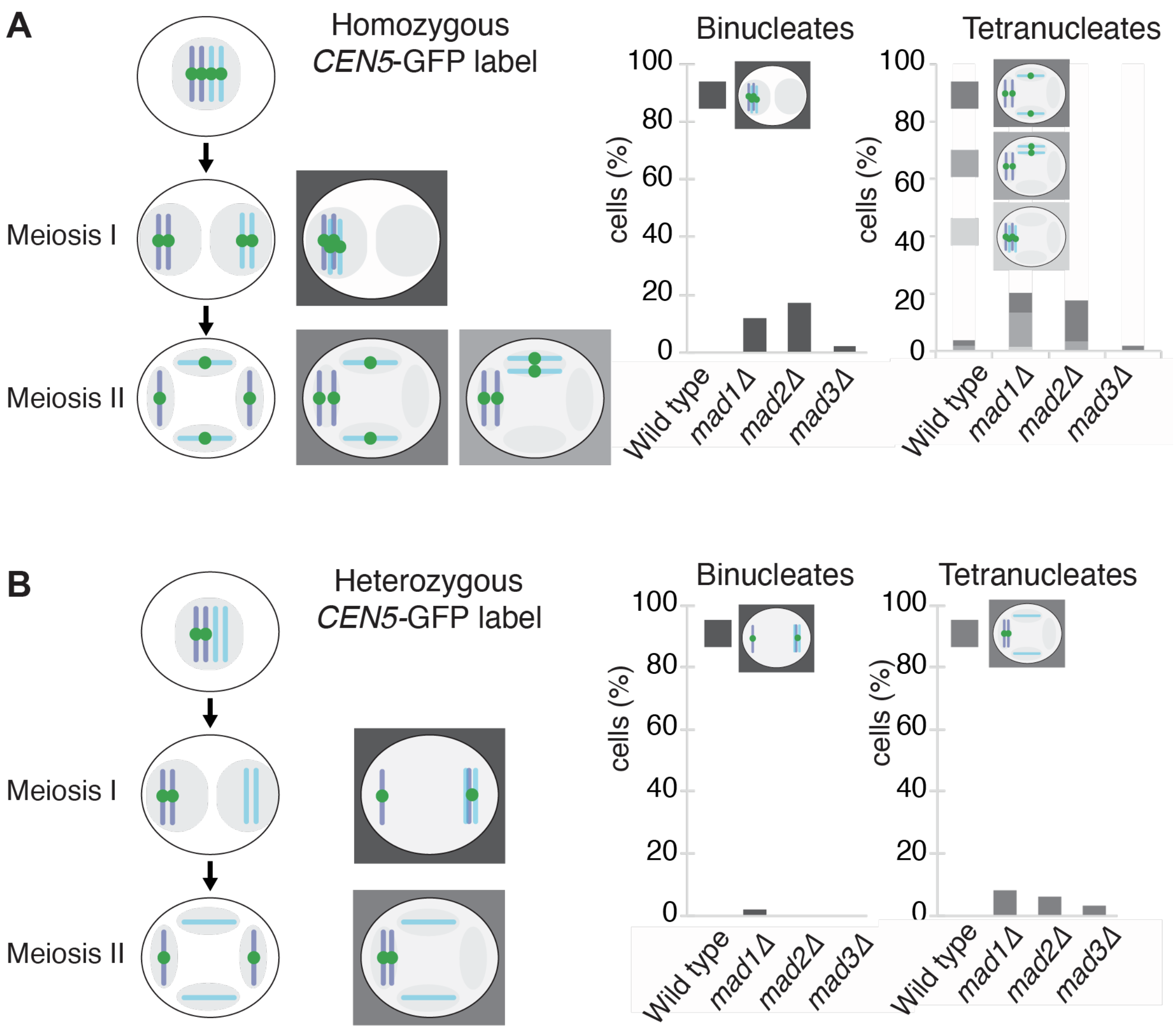

Figure S2 Mad2 is more critical than Mad3 for homolog segregation. Related to Figure 1.

(A) Analysis of meiosis I and II segregation in *mad1Δ, mad2Δ* and *mad3Δ* cells carrying GFP label on both copies of chromosome V. Strains carrying homozygous *CEN5-GFP* were induced to sporulate, fixed at hourly intervals and counter-stained with DAPI. The pattern of chromosome segregation as shown in the schematic (left) was scored in 100 binucleate and 100 tetranucleate cells (right). (B) Analysis of meiosis I and II segregation in *mad1Δ, mad2Δ* and *mad3Δ* cells carrying GFP label on one copy of chromosome V. Strains carrying heterozygous *CEN5-GFP* were induced to sporulate and fixed at hourly intervals. The pattern of chromosome segregation as shown in the schematic (left) was scored in 100 binucleate and 100 tetranucleate cells (right).

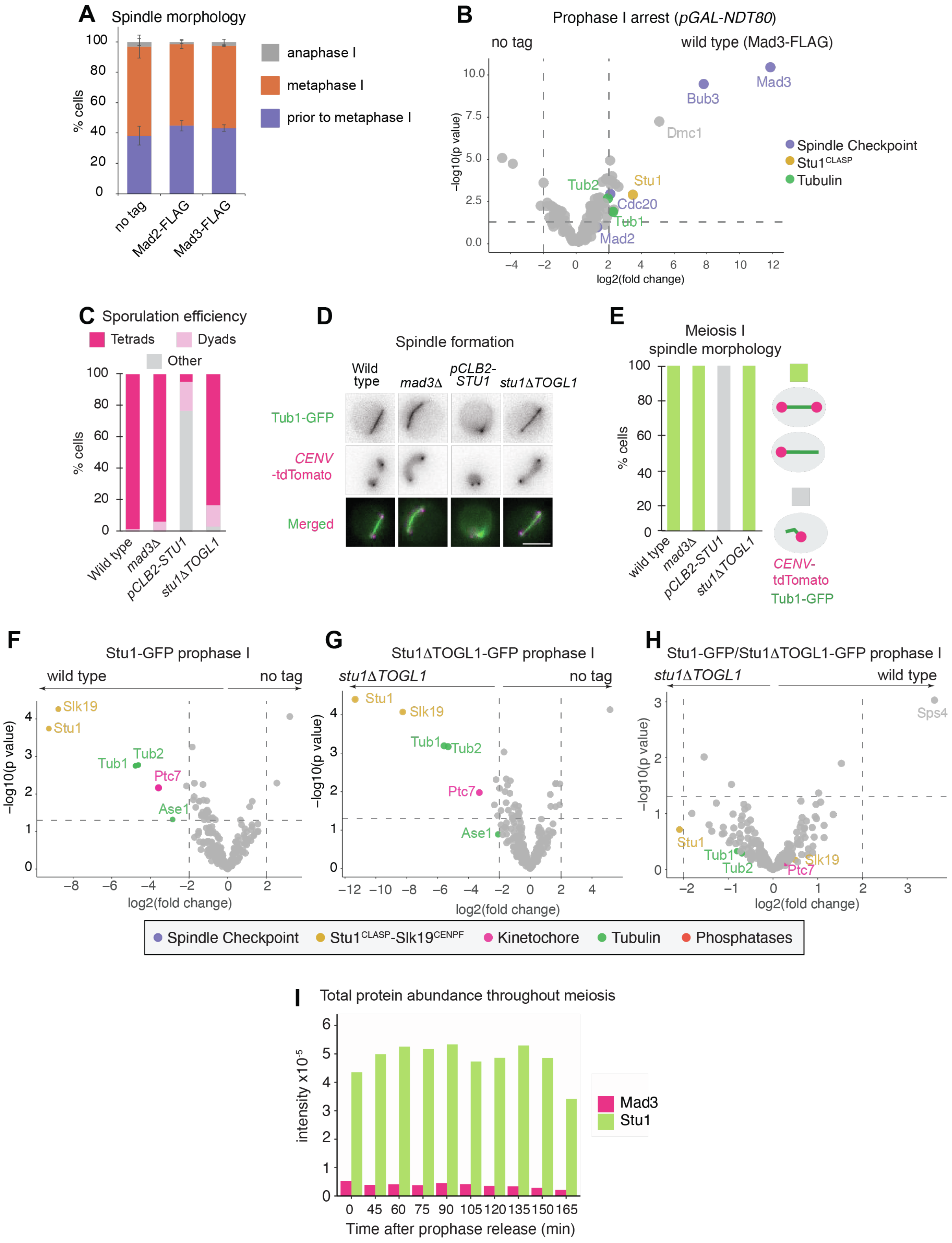

Figure S3 A subset of Stu1^{CLASP} interacts with Mad3^{BUBR1} in meiotic prophase but its TOGL1 domain is not required for association with tubulin, Slk19 or kinetochores, or for spindle formation. Related to Figure 2. (A) Confirmation that cells harvested for Mad2-FLAG and Mad3-FLAG shown in Figure 2A-C were in prometaphase/metaphase I. Spindle morphology after anti-tubulin immunofluorescence was scored in 200 cells of each replicate.

Mean of three biological replicates with error bars indicating standard deviation. (B) Stu1^{CLASP} associates with Mad3 in meiotic prophase I (*pGAL-NDT80* arrest). Volcano plots after mass spectrometry showing the relative enrichment of proteins immunoprecipitated with Mad3-FLAG in wild type vs no tag. Data shown is from the same experiment as shown in Figure 2G and H. The absence of a coloured dot for a kinetochore protein, phosphatase or tubulin in the volcano plot means that it was not detected in this experiment. (C) Stu1, but not its TOGL1 domain, is required for sporulation. The percentages of tetrads and dyads produced 72 h after inducing sporulation was scored for 200 cells of the indicated genotypes. (D and E) Stu1, but not its TOGL1 domain, is required for spindle formation during meiosis I. Strains of the indicated genotypes and carrying *CEN5-tdTomato* and *GFP-TUB1* were induced to sporulate and live imaged. Representative images (D; Scale bar = 5μm), together with the scoring of spindle morphology (E) in anaphase I cells (*n*=54 wild type; *n*=50 *mad3Δ*; *n*=50 *pCLB2-STU1*; *n*=56 *stu1Δtogl1*). Note that anaphase I spindles were not observed in *pCLB2-STU1* cells. (F-H) Tubulin and Slk19 are highly enriched in Stu1 immunoprecipitates independently of the TOGL1 domain. Stu1-GFP or Stu1ΔTOGL1-GFP anti-GFP immunoprecipitation followed by mass spectrometry was performed from cells harvested in a prophase I arrest (*GAL-NDT80*) Volcano plots show relative enrichments from three biological replicates and Log_2(Fold Change) between conditions is shown with corresponding p values. Dashed line indicates Log_2(Fold Change) = |2|. (F) Wild type Stu1-GFP vs no tag, (G) Stu1ΔTOGL1-GFP vs no tag and (H) Stu1-GFP vs Stu1ΔTOGL1-GFP. (I) Total abundance of Mad3 and Stu1 at prophase (t=0) and in 15 min intervals after prophase release encompassing the meiotic divisions. Data is the mean of two replicates and was plotted from[20]. See also Table S1.

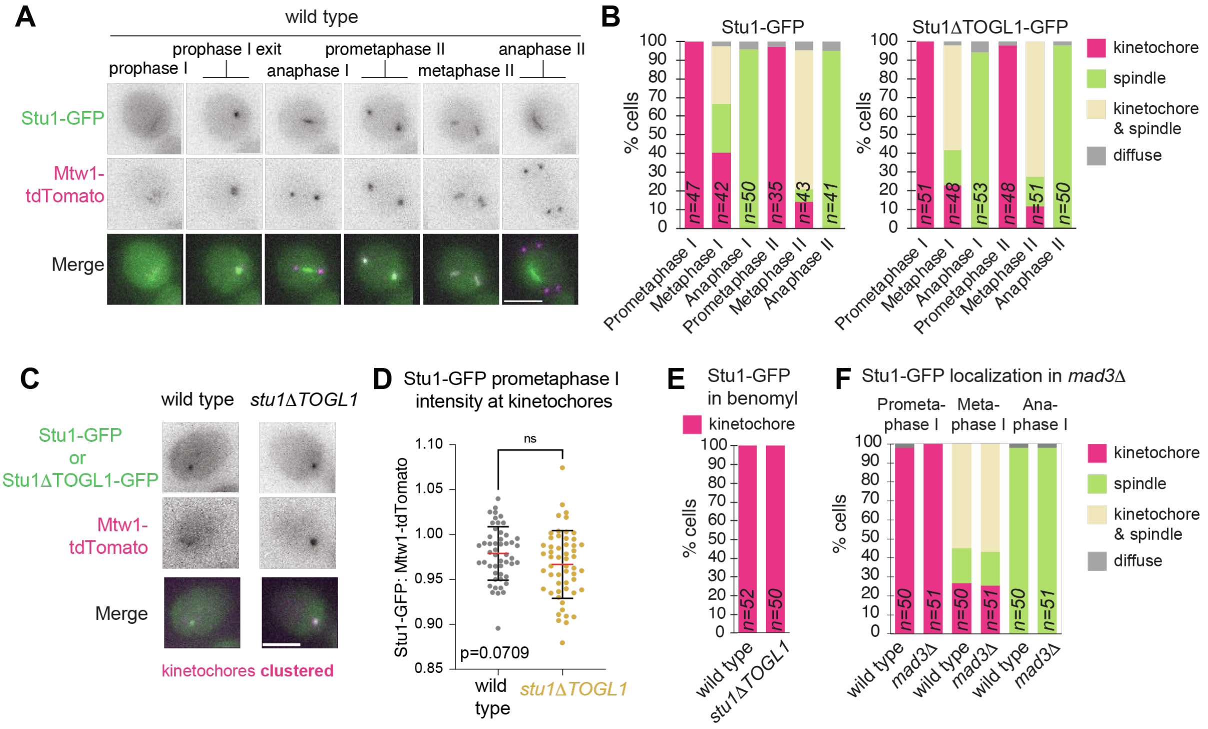

Figure S4 Stu1 kinetochore and spindle localization does not require the TOGL1 domain or *MAD3*. Related to Figure 2.

(A-E) Cells where *STU1* is replaced by either Stu1-GFP (wild type) or Stu1ΔTOGL1-GFP (*stu1ΔTOGL1*) were induced to enter meiosis and live imaged in the presence of DMSO (A-D) or benomyl (E). (A) Representative images of Stu1-GFP in the indicated meiotic stages. (B) Quantification of Stu1 localization at the indicated stages. (C and D) The ratio of intensity of GFP vs tdTomato signal was determined upon kinetochore clustering at prophase exit. (C) Representative images. (D) Stu1ΔTOGL1-GFP intensity is not significantly reduced at kinetochores compared to Stu1-GFP intensity. Red line indicates mean intensity, bars represent 95% CI. 50 cells were measured for each condition. p=0.709 Mann-Whitney test. (E) Quantification of Stu1-GFP and Stu1ΔTOGL1-GFP localization at kinetochores following benomyl treatment. (F) Mad3 is not required for Stu1 localization to kinetochores or spindles. Live cell imaging of wild type and *mad3Δ* cells undergoing meiosis was quantified as in B. Scale bars in A and C = 5μm.

**Mad3-FLAG** vs no tag in prometaphase I (Figure 2A)	
Bub3	Ptc3
Cda1	Ptc7
Cdc16	Rts1
Cdc20	Slk19
Cft1	Stu1
Dmc1	Tep1
Mad2	Tub1
Mad3	Tub2
Mpm1	Tub3
Mtc1	

**Mad2-FLAG** vs no tag in prometaphase I (Figure 2B)	
Aro4	Ndc80
Bub1	Rqc2
Bub3	Sec8
Cdc20	Snu13
Kre28	Spc105
Mad1	Ste23
Mad2	Stu1

**Mad3-FLAG** vs Mad2-FLAG in prometaphase I (Figure 2C)	
Bub3	Ptc7
Cdc16	Rpt4
Dmc1	Slk19
Dnm1	Spo22
Emc11	Stu1
Mad3	Tub2

Fig 1G: Mad3-FLAG vs **Mad2-FLAG** in prometaphase I (Figure 2C)	
Bub1	Rps1B
Mad1	FRS2
Mad2	YKL156W

Mad3-FLAG in **wild type** vs *mad2Δ* in metaphase I arrest (Figure 2D)
Cft1
Slk19
Rpl38

Mad3-FLAG in **wild type** vs *stu1ΔTOGL1* in prophase I arrest (Figure 2G)
Stu1

Mad3-FLAG in **wild type** vs *pCLB2-STU1* in prophase I arrest (Figure 2H)
Stu1

**Mad3-FLAG** vs no tag in prophase I arrest (Figure S3B)	
Bub3	Rim4
Bmh2	Rpn1
Cdc20	Rpt3
Cor1	Ssa3
Dmc1	Sse1
Erg10	Stu1
Hsp60	Tub1
Mad3	

**Stu1-GFP** vs no tag in prophase I arrest (Figure S3F)	
Ase1	Stu1
Pfk2	Tub1
Ptc7	Tub2
Slk19	

**Stu1ΔTOGL1-GFP** vs no tag in prophase I arrest (Figure S3G)	
Bmh2	Slk19
Pfk2	Stu1
Pgi1	Tub1
Ptc7	Tub2

Table S1 List of proteins significantly enriched in mass spectrometry datasets. Related to Figure 2 and Figure S3.
